# Evolution of protein-protein interaction networks in yeast

**DOI:** 10.1371/journal.pone.0171920

**Published:** 2017-03-01

**Authors:** Andrew Schoenrock, Daniel Burnside, Houman Moteshareie, Sylvain Pitre, Mohsen Hooshyar, James R. Green, Ashkan Golshani, Frank Dehne, Alex Wong

**Affiliations:** 1 School of Computer Science, Carleton University, Ottawa, Canada; 2 Department of Biology, Carleton University, Ottawa, Canada; 3 Department of Systems and Computer Engineering, Carleton University, Ottawa, Canada; King's College London, UNITED KINGDOM

## Abstract

Interest in the evolution of protein-protein and genetic interaction networks has been rising in recent years, but the lack of large-scale high quality comparative datasets has acted as a barrier. Here, we carried out a comparative analysis of computationally predicted protein-protein interaction (PPI) networks from five closely related yeast species. We used the Protein-protein Interaction Prediction Engine (PIPE), which uses a database of known interactions to make sequence-based PPI predictions, to generate high quality predicted interactomes. Simulated proteomes and corresponding PPI networks were used to provide null expectations for the extent and nature of PPI network evolution. We found strong evidence for conservation of PPIs, with lower than expected levels of change in PPIs for about a quarter of the proteome. Furthermore, we found that changes in predicted PPI networks are poorly predicted by sequence divergence. Our analyses identified a number of functional classes experiencing fewer PPI changes than expected, suggestive of purifying selection on PPIs. Our results demonstrate the added benefit of considering predicted PPI networks when studying the evolution of closely related organisms.

## Introduction

Physical and genetic interactions are fundamental to the understanding of cell biology [1–3]. Proteins seldom work in isolation, but rather function via their interactions with other proteins through both transient interactions, such as those that mediate phosphorylation, and more permanent interactions, like the formation of protein complexes. As a result, changes in protein-protein interactions (PPIs) can have important consequences for organismal fitness: several disease causing mutations are known to disrupt PPIs [4, 5], and single nucleotide polymorphisms (SNPs) associated with a number of diseases tend to occur in sites predicted to mediate PPIs [6].

Given the potential importance of PPIs to fitness, there is increasing interest in understanding the evolution of protein-protein and genetic interaction networks, as well as in clarifying the role of network architecture in determining the pace and trajectory of molecular evolution [3, 7–16]. For example, in comparing genetic interaction maps between budding yeast and fission yeast, Roguev *et al*. found that genetic interactions within functional modules are highly conserved, though cross-talk between modules varies between these two species [12]. In another study, Knight *et al*. demonstrated that the pleiotropic effects of a single adaptive mutation can be understood, at least in part, by its effects on protein co-regulatory networks [10].

One barrier to the study of network evolution is a lack of comparative data. While high-quality whole genome sequences may now be generated for multiple related organisms (e.g. [17, 18]), experimental determination of genetic or PPI networks is difficult and often not feasible in many systems. For example, standard approaches for the determination of genetic interaction networks in yeast [2, 12] and *E*. *coli* [19, 20] require the availability of large libraries of single gene knockouts or overexpression constructs, which are then crossed in all pairwise combinations, generating tens of thousands to millions of strains whose growth rates must be assayed. Genome scale genetic interaction analysis is becoming more prevalent and will likely expand to several new systems over the coming years [21], but remains a challenging undertaking. Similarly, studying PPI networks through affinity-purification or yeast two-hybrid approaches requires extensive human effort, facilities, and expertise [22–25]. In addition, such endeavors also yield significant false positive/negative rates because of inherent limitations in the methodology and sheer technical complexity. As such, large-scale, high-quality network data appropriate for comparative analyses are available in only a very few cases (e.g. [26]).

In addition to the technical hurdles posed by experimental determination of networks in multiple species, conceptual and analytical challenges complicate the interpretation of such large scale comparative data. For example, Ideker and Krogan [3] have encouraged the development of statistical methods for the analysis of differential network analysis, noting in particular the high variance associated with comparing interaction measures under multiple conditions (including multiple species). Additionally and importantly, it can be difficult to formulate null hypotheses concerning network evolution. Null hypotheses for DNA sequence evolution are widely used in sequence-based evolutionary studies [27–29], but comparable null models are not readily available for network evolution. Nonetheless, in order to identify conserved PPIs, we must be able to provide null expectations concerning how much change is expected through mutation alone. Only against the backdrop of a well-formulated null hypothesis can we identify portions of a network that are particularly well conserved, or that evolve very rapidly.

Here, we take a novel approach to the study of PPI network evolution, by comparing computationally inferred PPI networks for five species of yeast: *Saccharomyces cerevisiae*, *S*. *paradoxus*, *S*. *bayanum*, *S*. *kudriavzevii*, and *S*. *mikatae*. *S*. *cerevisiae*, a key model organism for genetics and the first eukaryote to be sequenced [30], has long had a high quality, well-annotated genome sequence and the best characterized proteome of any non-viral species [31]. High quality whole genome sequences for the latter four yeasts, as well as gene annotations and alignments, were recently made available by Scannell *et al*. [18]. As such, this set of closely related yeasts provides a powerful system for studying genome evolution, including the evolution of PPI networks.

Our computational inference makes use of the Protein-protein Interaction Prediction Engine (PIPE), an algorithm that predicts PPIs on the basis of protein primary sequence only [32–36]. PIPE breaks query proteins into short overlapping polypeptide segments and searches within a list of known and experimentally verified PPIs to find similar segments. The frequency of co-occurrence for a pair of polypeptide sequences from the query proteins (one sequence from each protein) that are found to be similar to a pair of sequences within known interacting proteins is considered evidence that the query proteins may interact. The decision threshold of this method can be tuned to have an extremely high specificity (99.95%), such that the predictions have relatively few false-positives. Our ability to achieve such high specificity is a distinguishing feature of PIPE [37], and is critical when one intends to examine millions of protein pairs (effectively testing millions of hypotheses). PIPE has been used to identify novel protein interactions, to discover new protein complexes, to predict novel protein functions [32–36], and to produce proteome-wide predicted interaction networks for *S*. *cerevisiae* [34], *Schizosaccharomyces pombe* [33], *Caenorhabditis elegans* [35] and *Homo sapiens* [36], among others.

While computational inference of PPI networks offers the advantages of speed and affordability in comparison to experimental approaches, the PIPE algorithm has the potential for bias when applied to comparative datasets. For the current study, the PIPE database consists of experimentally determined PPIs from *S*. *cerevisiae*. Because PPIs are then predicted on the basis of short polypeptide sequence pairs within the query proteins that reoccur in a number of known interacting proteins, predictions may be more accurate for species closely related to *S*. *cerevisiae* than they are for more distantly related species. We do note that interactions from one species can be used to predict interactions in another, even using distant relatives such as human and yeast. However, within-species interactions have been found to be more accurate [33].

**Fig 1:**
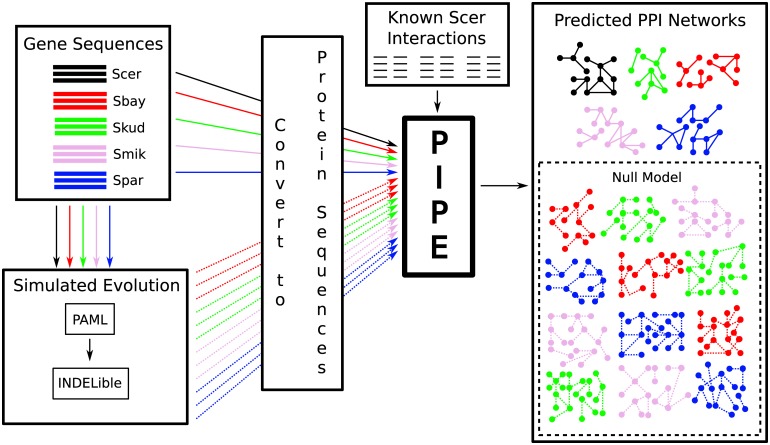
An overview of the computational process used to infer PPI networks for each of the 5 yeast species, and to generate the simulated null model. Molecular evolutionary parameters were inferred under the M0 model in PAML, and were used to generate simulated datasets using INDELible. PIPE was used to infer PPI networks for both the real and simulated datasets.

We propose that two key problems—potential bias associated with using the PIPE algorithm for cross-species predictions, and the challenge of formulating a null hypothesis for network evolution—can be addressed using a common approach. In both cases, expectations must be formulated with respect to the effects of mutations on the inferred PPI network: in the case of controlling for bias associated with PIPE, how do random mutations affect PIPE’s inferences? And, with respect to a null hypothesis for network evolution, how much change in a PPI network is expected given random mutation (i.e., mutations that are random with respect to PPIs)? We provide null expectations for changes in the inferred PPI network using simulated proteomes from the four non-*cerevisiae* yeasts. In the simulated proteomes, the locations of substitutions (both point mutations and insertion-deletions) are random with respect to PPIs. This is equivalent to assuming that natural selection does not operate for or against mutations that modify PPIs, as is appropriate in a null model. The rates and types of substitutions, however, are modeled on the real sequence data. As such, the simulated datasets provide a baseline expectation for how many changes we expect to infer using the PIPE algorithm, given mutation but no natural selection on PPIs. Using simulations, we propose to both mitigate bias associated with PIPE, as well as to provide a null hypothesis against which selection can be inferred. An overview of this process is illustrated in Fig 1.

Given the experimental and conceptual challenges posed by the study of network evolution, the computational approaches proposed in this study should provide powerful tools for describing the evolution of PPI networks, and for making inferences concerning the action of natural selection at the network level. Importantly, in studying the evolution of PPIs and their networks, we gain additional evolutionary insights that are not apparent from the study of sequence evolution in individual genes.

## Materials and methods

### Sequences and gene-centered analyses

Multiple sequence alignments for five species of yeast, *S*. *cerevisiae*, *S*. *paradoxus*, *S*. *bayanum*, *S*. *kudriavzevii*, and *S*. *mikatae*, were obtained from Scannell *et al*. [18]. We used alignments annotated as “high quality”, corresponding to 4,179 protein coding genes. This set of high-quality alignments corresponds to about two-thirds of genes in the *S*. *cerevisiae* genome; gene ontology (GO) categories represented in this set of genes are given in S1 Table

Scannell *et al*. applied standard molecular evolutionary analyses implemented in PAML (Phylogenetic Analysis by Maximum Likelihood [29]) to the full set of high-quality alignments. Gene-averaged ω (the ratio of nonsynonymous to synonymous changes per site, dN/dS) was estimated under the M0 model; we considered the set of genes falling in the lowest 10^th^ percentile of ω to be highly conserved (corresponding to those genes with ω < 0.0304). Positive selection was inferred using the standard M8 vs. M7 comparison. Here, the null model M7 allows variation in ω according to a beta distribution, but does not allow any sites with ω > 1. The alternative model M8 adds an additional class of sites with ω > 1; the difference in -2ΔlnL between M7 and M8 is expected to follow a χ^2^ distribution with two degrees of freedom, allowing for statistical hypothesis testing. For the purposes of comparing sets of rapidly evolving genes identified using sequenced-based and network-based methods, we applied a non-conservative threshold of *P* < 0.05 to identify positively selected genes.

### The sequential PIPE algorithm and MP-PIPE

For a given organism, PIPE relies on a database of known and experimentally verified protein interactions to predict novel PPIs. The database represents an interaction graph *G* where every protein corresponds to a vertex in *G* and every interaction between two proteins *X* and *Y* is represented as an edge between *X* and *Y* in *G*. The remainder of this section outlines how, for a given pair (*A*, *B*) of query proteins, PIPE predicts whether or not *A* and *B* interact.

In the core PIPE algorithm, protein *A* is sequentially examined using overlapping fragments of 20 amino acids. This can be thought of using a sliding window of size 20 across protein *A*. For each fragment *a*_*i*_ of *A*, where 1 < = *i* < = |*A*| - 20 + 1, we search for fragments “similar” to *a*_*i*_ in every protein in the graph *G*. A sliding window of size 20 is again used on each protein in *G*, and each of the resulting protein fragments is compared to *a*_*i*_. For each protein that contains a fragment similar to *a*_*i*_, all of that protein’s neighbors in *G* (interaction partners) are added to a list *R*. To determine whether two protein fragments are similar, a score is generated using the PAM120 substitution matrix. If the similarity score is above a tuneable decision threshold, then the fragments are said to be similar. In the next step of the PIPE algorithm, protein *B* is similarly examined using overlapping fragments *b*_*j*_ of size 20 (1 < = *i* < = |*B*| - 20 + 1) and these fragment are compared to all (size 20) fragments of all proteins in the list *R* produced in the previous step. We then create a result matrix of size *n* x *m*, where *n* = |*A*| and *m* = |*B*|, and initialize it to contain zeros. For a given fragment *a*_*i*_ of *A*, every time a protein fragment *b*_*j*_ of *B* is similar to a fragment of a protein *Y* in *R*, the cell value at position (*i*, *j*) in the result matrix is incremented. The result matrix indicates how many times a pair of fragments (*a*_*i*_, *b*_*j*_) co-occurs in protein pairs that are known to interact. It is based on this matrix that the query proteins are predicted to interact or not. The MP-PIPE system is a massively parallel, high throughput protein-protein interaction prediction engine and is the first system capable of scanning the entire protein interaction network of complex model organisms [35]. Although other PPI prediction methods exist, they all suffer from one or more drawbacks which only allow them to investigate a small portion of a given interactome and do not allow them to process all possible protein pairs. These drawbacks include a reliance on unavailable or unreliable biological data (evolutionary history, domains, 3D structure, etc.), high computational complexity leading to excessive run times, and unacceptably high error rates, among others [36].

### Generation and analysis of a null model of PPI network evolution

Phylogenetic methods were used to simulate proteomes for each of the four non-*cerevisiae* organisms (*S*. *paradoxus*, *S*. *bayanum*, *S*. *kudriavzevii*, and *S*. *mikatae*), using substitution and insertion-deletion (indel) parameters inferred from the true dataset. Scannell *et al*. [18] reported high-quality alignments for 4179 genes with strict orthologs in each of five yeast species, and provided nucleotide substitution parameters, on a gene-by-gene basis, under the M0 model in PAML [29]. Furthermore, for each gene we inferred the number and size of indels across the five-species phylogeny using a simple parsimony model. This distribution was used to estimate parameters of a power-law function using the power.law.fit() in R [38]. Nucleotide and indel parameters were then used to generate simulated gene sequences using INDELible [39]. For each gene, 100 simulated datasets were generated. Here, the *S*. *cerevisiae* gene sequence was kept constant (i.e., same sequence as the true data), and sequences for the other four species were simulated taking the *S*. *cerevisiae* sequence as the root, using the tree (*S*. *bayanus*, *S*. *kudriavzevii*, (*S*. *mikatae*, (*S*. *paradoxus*, *S*. *cerevisiae*))). *S*. *cerevisiae* is of course not the true root of the tree, but our approach is justifiable given the use of reversible substitution models.

For each of the simulated proteomes, *S*. *cerevisiae* interactions extracted from BioGRID [40] (containing 74,608 interactions) were used to infer the PPI network. MP-PIPE was run on all 400 simulated proteomes, as well as the five “real” proteomes. The predicted interactions from these five real proteomes, denoted Scer, Sbay, Skud, Smik and Spar, are available in S2, S3, S4, S5 and S6 Tables, respectively. To estimate the predictive performance of MP-PIPE, Leave-One-Out Cross-Validation (LOOCV) tests were done. To do this for a given organism, a set of positive (those known to interact) and negative (those expected not to interact) protein pairs are needed. In this case, the LOOCV tests were carried out in *S*. *cerevisiae* due to the lack of known interactions in the other yeast strains. The tests were carried out using the 74,608 known interactions and a set of 100,000 randomly chosen protein pairs (ensuring they do not occur in the known set) as the negative set. It has been shown in other studies that this is the most appropriate way to create bias-free negative interaction sets due to the lack of experimentally determined negative interactions [41, 42]. The known interactions are then tested, one at a time, by removing them from the MP-PIPE database and then attempting to predict if the proteins interact or not. These results are then combined with the predictions on the negative set. When this combined list is sorted by their prediction score, one can set a decision threshold and see, based on the LOOCV test results, what the achieved sensitivity (true positive rate) and specificity (true negative rate) were. In our case, since the density of PPI networks is expected to be very low, a decision threshold achieving an extremely high specificity is needed to minimize the number of false positives produced. A decision threshold which achieved a specificity of 99.5% and a sensitivity of 29.14% was chosen. To remain consistent across all predictions made, this decision threshold was used across all strains, both real and simulated. For more details on how these LOOCV tests are conducted see [36].

### Quantification of changes in the PPI network

**Fig 2:**
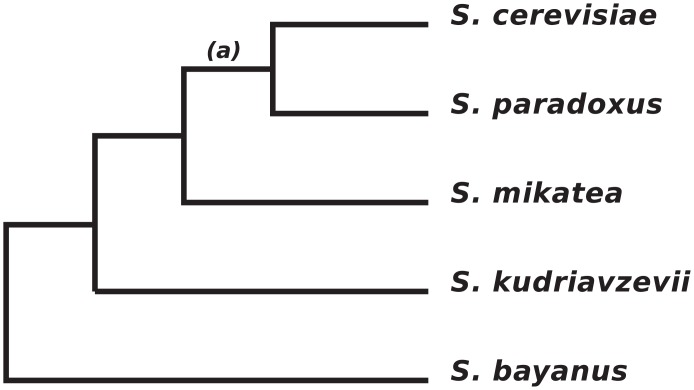
Phylogeny of the five yeast species studied here.

If a particular interaction is inferred to be absent in a single species then the simplest assumption is that a single change—a loss of interaction—has occurred along the phylogenetic tree. However, if an interaction is predicted to be absent in more than one species, then it may be necessary to infer more than one change, depending on which species are missing the interaction. For example, in Fig 2, an interaction present in all species except for *S*. *cerevisiae* and *S*. *paradoxus* would require only a single change of state, a loss of interaction along the branch ancestral to these two species (branch marked “a”). By contrast, an interaction present in all species except *S*. *mikatae* and *S*. *kudravzevii* would require two changes: either two losses, or a loss and a gain. Thus, for each PPI inferred to be present in at least one species, we inferred by parsimony the minimum number of changes (losses or gains of interaction) required to explain the observed pattern of presence/absence of that interaction. For a binary character in five taxa, there are thirty-two possible combinations of states, with at most three changes required for any given pattern. For a given interaction between proteins *i* and *j*, we define the quantity a_ij_ as the number of inferred changes in interaction state for the pair across the phylogenetic tree. We note that maximum-likelihood methods could also be used to infer the number of state changes, but given the small number of taxa and relatively shallow tree, parsimony should yield comparable results.

Since many proteins in the interaction network may have more than one interaction partner, for every protein *i* with *n* total interaction partners, we calculate the total number of changes in PPIs across the phylogeny (*γ*_*i*_) as:

$$\gamma_{i}=\overset{n}{\underset{j=1}{\sum }}a_{ij}$$

*γ*_*i*_ is thus the total number of inferred PPI changes across the phylogenetic tree for a given protein in the network.

Using PIPE-predicted PPIs, γ was calculated for every protein in the real dataset, as well as in all 100 simulated datasets. The distribution of γ from the simulated datasets was then used as a null distribution to evaluate whether an observed true γ was unusual, i.e., particularly high or particularly low.

### Species-specific analyses

In addition to changes in the PPI network across the entire tree (as quantified by γ above), we investigated lineage-specific patterns of PPI evolution for each of the non-*cerevisiae* species. For a given species, we identified interactions inferred to be present in the real proteome, but not in any of the 100 simulated proteomes, for that species.

### Gene Ontology (GO) analysis

For sets of proteins identified as having either conserved or rapidly evolving PPIs across the phylogeny (low or high *γ*, respectively), we carried out GO term enrichment analysis. The Gene Ontology [43] data used was the *S*. *cerevisiae* data, version 2013-08-31. For a given GO term we would calculate its hypergeometric *p*-value using the four following numbers: the number of proteins in the current set associated with the given GO term, the total number of proteins in the current set, the number of proteins associated with the given GO term in the global set of proteins, and the total number of proteins in the global set.

For the sets of PPIs found to be present in the “real” interactomes of a particular species, but absent in the simulated interactomes, a modified GO analysis was conducted. Here, we were interested in GO enrichment for pairs of proteins, and as such we modified the conventional procedure for identifying enriched GO terms. For each interaction in a given set, the tags that both proteins had in common were identified. Once this was done for each pair in the set, we are left with a set of common GO terms as well a count of how many times they were common to an interaction in the overall set. A p-value could then be calculated for each GO term given the number of times it was common to an interaction, the number of interactions in the set, the total number of possible interactions within the entire proteome and the number of these pairs that share the term in question. Specific rules were used to filter the GO enrichment results. In most cases, the following rules were applied:

- p-value less than 0.05
- must be a process GO term
- at least 3 of the proteins in the set must be associated with the term (remove tags with only 1 or 2 associated proteins)

These rules were used in all cases with the exception of protein cluster analysis. Since clusters are much smaller sets of proteins compared to the other sets, the third rule was amended to allow for tags only associated with 2 proteins in the cluster. On top of this, for the cluster analysis, function GO terms were also investigated.

### OSLOM cluster analysis

Dense subgraphs of an interactome can represent functionally-related proteins or proteins that interact to form protein clusters. OSLOM (Order Statistics Local Optimization Method) [44] is a computational tool used to find clusters within a graph. OSLOM identifies statistically significant clusters with the use of a null model consisting of a random graph with the same node degree distribution as the input graph. When OSLOM is building a cluster and is considering adding a given node, it consults the null model. If the node shares many more edges with the cluster in the original graph than would be expected under the null model it can be included into the cluster, as the connections between the node and the cluster are unexpectedly strong. This process is run until no further nodes can be added to the cluster. The entire process is then repeated using a different random point in the graph until the entire graph is covered, resulting in a set of statistically significant, and potentially overlapping clusters. OSLOM was run on all of the MP-PIPE-produced interactomes to give a set of overlapping clusters within each interactome. From here, clusters from different interactomes can be compared by counting how many proteins they have in common. A similarity score was defined between two clusters as the number of proteins the two clusters have in common divided by the size of the larger of the two clusters. Using this as a scoring function will always result in a score between 0 and 1.

Similar to the species-specific analyses, clusters that existed in the real dataset, but that did not exist in any of the 100 simulated interactomes, were sought. To determine if a given cluster was unique to the real data, it was compared to all clusters in all 100 simulated interactomes. If it was determined not to match any of these clusters (here two clusters are considered a match if their similarity score is greater than or equal to 0.7) then it was deemed unique to the real data and, therefore, of potential functional importance.

For each set of putatively functionally important clusters (one set for each species), GO term enrichment analysis was carried out to determine which GO terms present in each cluster were statistically significant.

## Results

### Changes in PPIs across the phylogeny

**Fig 3:**
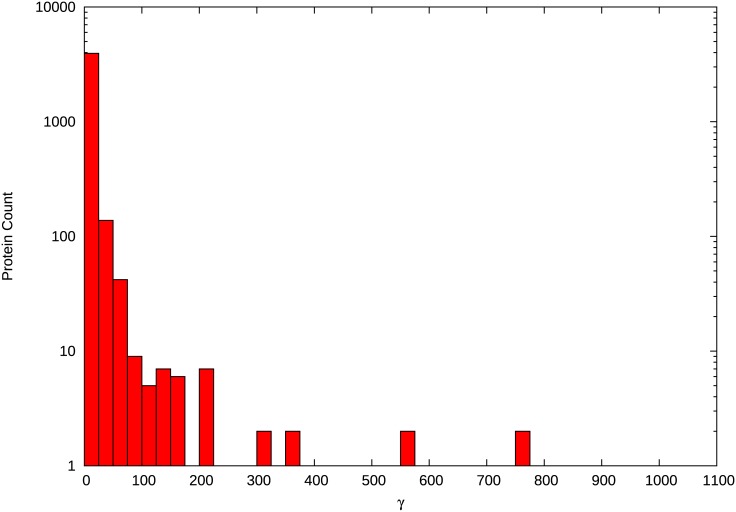
Distribution of the change in protein-protein interaction across the phylogeny. The distribution of γ, which represents the total number of interaction changes (gains or losses) over the entire phylogeny. The majority of proteins experience relatively few changes in interaction across the phylogeny with a small number of proteins experiencing many changes.

Phylogeny-wide changes in PPI profiles were estimated for 4179 proteins with high quality alignments from the 5-species yeast dataset of Scannell *et al*. [18]. For each protein, we estimated γ, which represents the total number of interaction changes (gains or losses) over the entire phylogeny (S7 Table). Median γ was 3.0, with a mean and variance of 10.06 and 2,001.74, respectively. The full distribution of γ is shown in Fig 3. It is clear that the bulk of proteins are inferred to experience very few PPI changes, but the long tail of the distribution suggests a subset of proteins experiencing many changes. This leads to the exceptionally high estimate of variance.

**Fig 4:**
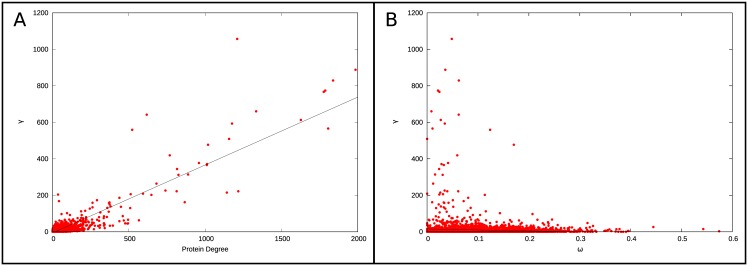
Comparing the change in protein-protein interaction to protein degree and rate of sequence change across the phylogeny. Comparison of changes in PPIs across the phylogeny (γ) to degree (A) or to rate of substitution across the phylogeny (ω) (B). A protein’s γ is correlated with its degree in the network (see regression line in panel A), but not with its overall rate of substitution.

A protein’s γ is correlated with its number of interactions in *S*. *cerevisiae* (i.e., its degree), such that proteins with many interactions tend to undergo more changes than proteins with few interactions (Kendall’s τ = 0.488, P < 2x10^-16^; Fig 4A). This correlation is expected since a protein with more PPIs has more opportunities to lose interactions than a protein with few interactions.

Notably, γ is not correlated with overall rates of protein sequence evolution (Fig 4B). No correlation is found when the rate of protein evolution is measured as the raw rate of non-synonymous substitution dN (τ = -0.012, P = 0.247), or when it is measured as ω, the ratio of non-synonymous to synonymous substitution rates (τ = -0.014, P = 0.175; ω and dN estimated under model M0 in PAML by Scannell *et al*. [18]). Thus, changes in our predicted PPI profile do not appear to be determined by overall changes in amino acid sequence and raises the possibility that additional information can be gleaned by examining changes in PPIs because of the unique properties of the PIPE algorithm.

### Comparison of molecular evolution using sequence- and PPI-based methods

We compared the set of proteins whose primary sequences are conserved across the 5-species yeast phylogeny with the set of proteins whose interactions are predicted to be conserved using the PIPE algorithm. In order to identify proteins whose interactions are highly conserved between species, we generated null distributions of γ via simulation. For each of 100 simulated datasets, primary sequences for all 4,179 proteins in the yeast PPI network were simulated using substitution and indel parameters estimated from the true dataset. PIPE was then used to infer changes in PPI networks for the simulated data. Table 1 summarizes the sizes of the predicted interactomes generated by PIPE on both the real and simulated proteomes of each species. Importantly, in the simulations, the locations of mutations are random with respect to PPIs, such that differences between the real data and the simulated data are potentially attributable to selection on sites that mediate PPIs. Indeed, consistent with purifying selection on sites mediating PPIs, the number of inferred interactions in the simulated proteomes was systematically lower than the number of inferred interactions in the real proteomes (Table 1).

**Table 1:** Number of interactions in the predicted interactomes for four yeast species, inferred from real and simulated datasets.

	*S*. *bayanus*	*S*. *kudrivzevii*	*S*. *mikatae*	*S*. *paradoxus*
# inferred interactions: real data	90,473	88,752	89,111	89,908
Avg size of simulated interactome	72,351.99	74,069.29	76,371.29	81,741.31
Min size of simulated interactome	71,549	73,082	75,226	80,682
Max size of simulated interactome	73,398	75,115	77,529	82,721
Median size of simulated interactome	72,292	74,047	76,371	81,751

**Fig 5:**
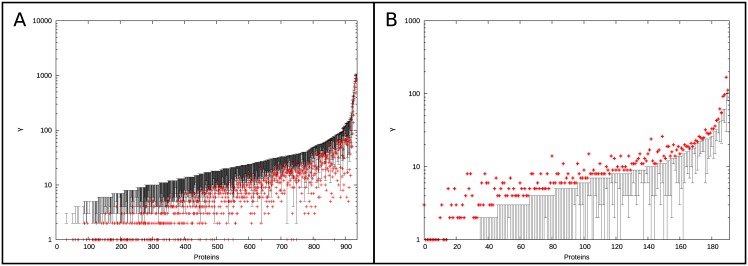
Proteins which experience a lower or higher number of changes in PPIs in the real data compared to the simulated interactomes. Proteins which experience a lower (A) or higher (B) number of changes in inferred PPIs in the real data in comparison to the simulated interactomes. Each protein’s real γ is plotted in red and the range of γ observed in the null model are plotted in black.

Proteins whose interactions are conserved were identified as those whose γ in the real dataset falls below γ in all of the 100 simulated datasets (for *P* < 0.01). Here, the simulated datasets provide a null distribution for inferred changes in PPIs owing to mutation alone; these changes will reflect both true losses or gains of interactions, and false positives—i.e., changes inferred by PIPE that do not reflect true changes. A reduced true γ in comparison to the simulated datasets provides evidence that natural selection maintains PPIs by selecting against interaction-altering mutations. 936 proteins—almost a quarter of the proteome—were identified by this criterion (Fig 5A).

We compared the set of proteins with conserved PPIs to those whose sequence is conserved. To identify sequence conservation, we used estimates of ω, the ratio of non-synonymous to synonymous substitution rates, from Scannell *et al*. [18]. Here, ω for each gene was estimated under the M0 model in PAML [29], which assumes a single value of ω for a given gene. We chose the 10% (418) of genes with the lowest ω as the set of proteins with the highest level of sequence conservation. The sets of proteins identified by sequence conservation and by conservation of PPIs shared 108 proteins; this overlap is slightly larger than expected by chance (one-sided Fisher’s exact test *P* = 0.047) (Table 2). Thus, there is only a weak overlap between proteins whose PPIs are inferred to be conserved and those whose primary sequence is conserved.

**Table 2:** A comparison of proteins and enriched GO processes that were identified through conservation or rapid evolution of sequence or of inferred PPIs.

	# Proteins	# Enriched GO Terms
Conserved		
Low ω	418	87
Low γ	936	101
Overlap	108	15
Rapidly changing		
High ω	123	7
High γ	191	12
Overlap	9	1

“Conserved” sequence refers to the 10% of genes with the lowest ω, while “positively selected” refers to 123 proteins inferred to be under positive selection. Unusually high or low γ includes proteins whose true γ is above (rapidly changing) or below (conserved) the distribution of γ from the simulated datasets. Enriched GO processes (p<0.05) are included. Details regarding the proteins/GO terms found in each set can be found in S8 Table.

While conserved proteins and PPIs reflect core processes that are maintained over the course of evolution by purifying selection, rapidly evolving proteins and PPIs may reflect diversifying selection over most or all of the phylogeny (e.g., [45]). We used data from Scannell *et al*. [18] for sequence-based inference of rapid evolution, with 123 rapidly evolving genes identified as those with evidence for positive selection in the M7/M8 comparison in PAML [29]. Proteins with evidence for rapid PPI evolution were identified as those whose γ in the real dataset exceeds γ in all 100 simulated datasets (Fig 5B); 191 such proteins were identified, with 9 proteins shared between the two datasets (Table 2). This overlap is not larger than that expected by chance (one-sided Fisher’s exact test *P* = 0.107).

In order to gain further insights into biological processes involving proteins with conserved or rapidly changing PPIs, we carried out GO analyses for the sets of proteins with low or high γ, as well as of those genes identified as conserved or as positively selected using sequence-based methods. Largely different sets of GO terms were identified as enriched using these two approaches (Tables 2–4). For example, 15 GO terms were shared by the sets of proteins whose sequences or PPIs were highly conserved, with 87 and 101 unique terms respectively. GO terms unique to proteins whose PPIs are conserved, or rapidly evolving, are given in Tables 3 and 4, respectively.

**Table 3:** Enriched GO terms for proteins with lower than expected γ not identified when analyzing slowly evolving protein sequences.

GO Term ID	GO Term Name	# Low γ proteins	*p*-value
GO:0006468	Protein phosphorylation	56	4.34E-12
GO:0016310	Phosphorylation	66	1.24E-07
GO:0032543	Mitochondrial translation	3	4.27E-07
GO:0002181	Cytoplasmic translation	40	1.52E-05
GO:0006810	Transport	175	2.38E-05
GO:0006897	Endocytosis	29	3.57E-05
GO:0007264	Small GTPase mediated signal transduction	22	1.17E-04
GO:0019236	Response to pheromone	14	1.45E-04
GO:0003333	Amino acid transmembrane transport	12	1.54E-04
GO:0006913	Nucleocytoplasmic transport	10	2.99E-04

Of the 87 unique GO terms identified with lower than expected γ not identified when analyzing slowly evolving protein sequences, the 10 with the lowest *p*-value are displayed.

**Table 4:** Enriched GO terms for proteins with higher than expected γ not identified when analyzing positively selected protein sequences.

GO Term ID	GO Term Name	# High γ proteins	*p*-value
GO:0008152	Metabolic process	3	0.0020
GO:0006351	Transcription, DNA-dependent	28	0.0079
GO:0010526	Negative regulation of transposition, RNA-mediated	3	0.0029
GO:0006355	Regulation of transcription, DNA-dependent	28	0.0035
GO:0006357	Regulation of transcription from RNA polymerase II promoter	10	0.0042
GO:0016310	Phosphorylation	3	0.0168
GO:0015031	Protein transport	9	0.0393
GO:0055085	Transmembrane transport	3	0.0406
GO:0000122	Negative regulation of transcription from RNA polymerase II promoter	8	0.0044
GO:0006366	Transcription from RNA polymerase II promoter	8	0.0186
GO:0006397	mRNA processing	12	0.0217

### Species-specific analyses

In order to characterize interactions that may be particularly important for lineage-specific biological processes, we identified interactions that are present in the predicted interactomes of each real dataset (i.e., in each species), but absent in all of the 100 simulated datasets for that species. Absence of such an interaction in the simulated data indicates that it is disrupted by mutation, at least according to PIPE predictions. These interactions may include those that are conserved in *S*. *cerevisiae*, and that are thus likely to have been maintained by purifying selection, as well as those that are predicted to be novel interactions in the non-*cerevisiae* species. The numbers of such interactions are as follows: *S*. *bayanum*: 7,552, *S*. *kudriavzevii*: 4,902, *S*. *mikatae*: 3,894, and *S*. *paradoxus*: 1,779.

Interactions identified as unique to a given real interactome with respect to the null model may represent functional interactions differentiating these yeast species. As such, we carried out GO enrichment analyses to identify processes that may be particularly important in each species. Relatively few GO process terms were enriched in each species, with 20, 2, 2, and 1 significant terms in *S*. *bayanus*, S. *kudriavzevii*, *S*. *mikatae*, and *S*. *paradoxus* respectively (S9 and S10 Tables).

We also investigated a fifth set of interactions, consisting of the intersection of the previously mentioned sets with interactions predicted in *S*. *cerevisiae*. This set of interactions can be thought of those interactions that were completely conserved across all five real interactomes but that did not occur in any of the 4x100 simulated strains. There were 662 of these phylogeny-wide conserved interactions which were subjected to GO enrichment analysis. Enriched GO terms for these conserved interactions were compiled in Table 5 and appear to be involved in five major cellular processes: cell cycle progression, DNA organization, signalling, lipid metabolism, and carbohydrate metabolism.

**Table 5:** Summary of the enriched GO Terms of the proteins participating in conserved PPIs.

General Process	GO Term ID	GO Term Name	# of PPIs	*p*-value
Cell Cycle	GO:0000070	Mitotic sister chromatid segregation	5	2.01E-11
	GO:0007049	Cell Cycle	13	1.81E-6
	GO:0007067	Mitosis	10	2.16E-11
	GO:0051301	Cell Division	12	2.15E-10
DNA Organization	GO:0007076	Mitotic chromosome condensation	3	6.14E-9
	GO:0007062	Sister chromatid cohesion	2	2.17E-6
	GO:0006310	DNA recombination	2	9.84E-4
	GO:0030261	Chromosome condensation	4	6.69E-13
Signalling	GO:0051276	Chromosome organization	3	1.21E-7
	GO:0000750	Pheromone-dependent signal transduction	7	2.18E-17
	GO:0007165	Signal transduction	7	6.48E-11
	GO:0007186	G-coupled receptor signalling pathway	3	5.76E-10
	GO:0031684	heterotrimeric G-protein complex cycle	2	8.60E-8
	GO:0019236	Response to pheromone	4	1.06E-8
Lipid Metabolism	GO:0006696	Ergosterol biosynthetic process	10	5.63E-25
	GO:0006629	Lipid metabolic process	13	1.49E-13
	GO:0006694	Steroid biosynthetic process	10	1.42E-24
Carbohydrate Metabolism	GO:0005975	Carbohydrate metabolic process	4	1.55E-5
	GO:0006098	Pentose-phosphate shunt	2	2.34E-5
Other	GO:0070058	tRNA gene clustering	3	5.76E-10
	GO:0006607	NLS-bearing substrate import into nucleus	2	3.61E-6
	GO:0006409	tRNA export from nucleus	2	3.61E-6

GO Term (process) enrichment grouped by category of proteins involved in interactions which were conserved across the 5 yeast species studied here. These interactions were also not present in any of the simulated interactomes. GO IDs were common to both proteins participating in the interaction.

### OSLOM cluster analysis results

In addition to the individual PPIs examined so far, higher levels of organization, e.g., protein complexes, may also be important for the evolution of PPI networks. As such, we sought to identify protein clusters that are potentially important in each lineage. For each interactome, clusters were identified as groups of proteins with more connectivity than expected by chance, using OSLOM. Conserved clusters were then identified as those that were absent from all one hundred simulated interactomes, indicating that they are lost easily due to mutation. These clusters were then compared to the clusters found in the interaction networks of the 100 simulated strains for each species, and we removed any clusters that were in both the real and simulated data. GO enrichment analysis was performed on the remaining clusters for each organism. The details of these clusters can be found in Table 6.

**Table 6:** Significant protein clusters and GO term enrichment analysis results found in each of the four non-*cerevisaie* species.

Species	# Significant Clusters	# Clusters with Enriched GO Terms	# Clusters Enriched with Unique GO Terms
*S*. *bayanus*	27	27	21
*S*. *kudrivzevii*	22	21	21
*S*. *mikatae*	14	14	13
*S*. *paradoxus*	14	14	13

A significant cluster is defined as one that occurs in the wild type interactome but in none of the 100 simulated interactomes for a given species. For the specific members of each cluster or for their associated GO terms, please see the S1 File.

## Discussion

The availability of high quality genome sequences and annotations from five members of the genus *Saccharomyces sensu strico* provides a unique opportunity to study the evolution of PPI networks. We have used the PIPE algorithm [32–36] to predict interactomes for all five species, and we provide a null model for PPI network evolution by simulation. We find evidence for extensive conservation of PPIs, as might be expected given the importance of PPIs for basic cellular functions. Notably, analysis of conserved and rapidly evolving PPIs offers a unique insight into the processes that remain important over evolutionary time, as well as into those processes that might contribute to adaptation in new environments.

Previous studies have compared networks from highly divergent systems, identifying core conserved pathways. However, these studies primarily examine the transcriptome either monitoring co-expression [46] or transcriptional regulatory factors [47] and typically compare distant relatives (i.e. *S*. *cerevisiae*, *E*. *coli*, *A*. *thaliana*, *C*. *elegans*, *D*. *melanogaster* and *H*. *sapiens*). Here, we investigate network evolution over a much shorter time scale, where the age of the *Saccharomyces sensu stricto* genus is about 20 million years [48].

### Analysis of the PPI network

As a first step in measuring changes in PPIs, we quantified the total number of changes in the PPI profile of each protein across the phylogeny (γ). The majority of proteins experienced very few changes in their interaction profiles, with a median of 3 losses or gains of interactions per protein. As expected, γ is strongly correlated with the number of PPIs in *S*. *cerevisiae* (degree), presumably due to the increased opportunity to lose/gain interactions in proteins which interact with a larger number of partners (Fig 4A). We identified 936 unique proteins that are inferred to experience fewer changes than expected in their interaction profiles across the phylogeny. This is not surprising as multiple PPI networks have been reported as being extremely conserved across both closely and distantly related phylogenies [19, 49–51]. However, given the species examined, our data provide unique insight into networks conserved over shorter evolutionary distances.

It might be expected that proteins which diverge rapidly at the sequence level would also diverge rapidly at the level of PPIs. Interestingly, our data do not support this expectation: γ is correlated with neither the raw rate of non-synonymous substitution *dN* (Kendall’s τ = -0.012, P = 0.247) nor with the ratio of non-synonymous to synonymous substitution rates ω (τ = -0.014, P = 0.175). Thus, changes in the predicted PPI profiles do not appear to be determined by overall changes in amino acid sequence. Rather, it is likely that predicted changes in PPIs are mediated by substitutions in small regions of a given protein. PIPE infers PPIs on the basis of short amino acid motifs, which often represent only a very small subsection of a protein sequence. The smallest motif analyzed by PIPE (20 amino acids in length) covers only 4% of the average total protein length of 467 amino acids in *S*. *cerevisiae* [52]. PIPE exploits these motifs, which mediate PPIs, to make its predictions. Therefore, amino acid changes outside of these predicted interaction sites will likely not have a large impact on the overall PPI prediction score.

Given that changes in PPIs do not correlate well with overall rates of sequence change, we investigated the functional classes of proteins showing highly conserved, or rapidly evolving, PPIs. GO analysis of proteins involved in conserved sets of interactions showed enrichment in five core biological processes: signal transduction, cell cycle progression, chromosome integrity/DNA repair, lipid metabolism, and transport (Tables 3 and 5). Previous studies have identified cell cycle progression, signal transduction, chromatin repair/recombination and transport are as highly conserved processes, using sequence conservation and empirical studies of protein complex [16, 53, 54]. Our results shed further light on the conservation of these processes. For example, a key chromosome remodelling protein and member of the condensing complex demonstrated a highly conserved interaction profile. Smc2, which reorganises chromosomes during mitosis and meiosis, maintained 29 interactions across the phylogeny, eight of which were with key participants in mitotic sister chromatin segregation including the master regulator of mitosis, Cdc28.

Our results also point to the conservation of processes and pathways that have not been previously highlighted, such as lipid metabolism (which was not identified as conserved in previous studies–[16, 54]). For example, Erg7, which catalyzes a step in the ergosterol biosynthetic pathway, maintained 21 interactions across all lineages, 15 of which were with proteins involved in lipid metabolism. Thus, these results suggest that network analysis on the basis of predicted PPIs provides a unique perspective on essentiality, and may help identify interactions which are fundamental across this phylogeny.

### Identification of putatively functionally important sets of PPIs

In our phylogeny-wide analysis of changes in the PPI network, we identified a number of GO terms that were enriched in the sets of proteins with low or high levels of PPI change. A number of these terms were not enriched when we examined sequence change alone (Tables 3 and 4), suggesting that additional insights can be gained by investigating PPI evolution.

For example, a total of 106 yeast genes are annotated with the “GO:0006468—protein phosphorylation” term, 56 of which were identified as undergoing significantly less change than the changes observed in the simulated datasets (GO enrichment: P = 4.34x10^-12^). We also identified GO:0016310 phosphorylation (1.24x10^-7^), GO:0046777 protein autophosphorylation (7.79x10^-3^), and GO:0006470 protein dephosphorylation (2.95x10^-2^) as enriched in this dataset, strongly suggesting conservation of PPIs amongst proteins involved in phosphorylation. Consistent with this finding, kinases contain highly conserved regions which are essential to protein function [55], and activation of protein kinases also appears to be highly conserved (e.g., [56, 57]).

The GO term “phosphorylation” (GO:0016310) is also over-represented amongst PPIs that change more rapidly than expected (Table 4). Different sets of phosphorylation-related proteins appear in low- and high-gamma set, with 3 phosphorylation-related proteins (all kinases) in the high gamma set, and 56 in the low-gamma set (55 of which are kinases). We were unable to find any features that distinguished the high- from the low-gamma sets (e.g., number of targets, localization, essentiality, or target type).

In addition to identifying PPIs that are conserved throughout the phylogeny, we investigated PPIs that may be particularly important to the biology of each individual species. For each non-*cerevisiae* interactome, we identified PPIs (or protein clusters) inferred in the real dataset, that were absent from all 100 simulated interactomes for that species. Absence of such interactions or clusters in the simulations suggests that they are susceptible to disruption via mutation, such that selection has maintained them in the real interactomes (S9 and S10 Tables).

For example, PPIs of proteins that help to facilitate amino acid transmembrane transport are conserved in the real *S*. *bayanus* interactome in comparison to the simulated interactomes (GO:0003333–9.0x10^-7^). We speculate that such PPIs may partially underlie cryotolerance and low temperature fermentation in *S*. *bayanus*: At low temperatures, transport of aromatic amino acids is impaired due to mechanical stress caused by increased membrane rigidity. Global metabolomic analysis has suggested that *S*. *bayanus* and *S*. *kudriavzevii* upregulate shikimate aromatic amino acid biosynthesis to respond to cold stress [58]. In response to the decreased rate of transmembrane amino acid transport, the tryptophan transporter Tat2p, and others, are overexpressed [59]. These results suggest that the response to reduced aromatic amino acid levels is counteracted by upregulation of amino acid transporter genes but not alterations to transporter PPIs.

Similarly, our analyses suggest mechanisms underlying alcohol sensitivity in *S*. *kudriavzevii*. This species has been to shown to be extremely sensitive to ethanol and is less tolerant than the other species examined in this study [60]. The genes most responsible for alcohol tolerance are associated primarily with cytoskeleton organization, biogenesis, and transport particularly involving the vacuole, peroxisome, and endosome [61]. Using OSLOM cluster analysis we identified interaction clusters in *S*. *kudriavzevii* that are enriched for GO terms not found in clusters from the other strains or in the null model that may help explain this hyper-sensitivity to ethanol. Cluster 113 is uniquely enriched for 11 GO IDs associated with cytoskeleton biogenesis and organization (Table 7) as well as the ID GO:0045324 late endosome to vacuole transport (p = 3.28x10^-6^). Other clusters were enriched for GO:0004026 alcohol O-acetyltransferase activity (p = 1.13x10^-4^) and GO:0030242 peroxisome degradation (p = 3.03x10^-5^). We propose that these unique interaction patterns in *S*. *kudrivzevii* which are enriched for terms associated with processes most important for alcohol tolerance help to explain this unique phenotype.

**Table 7:** Enriched GO terms in *S*. *kudriavzevii* cluster 113.

GO Term ID	Go Term Name	# Associated Proteins in Cluster	# Associated Proteins in Proteome	*p*-value
GO:0030472	mitotic spindle organization in nucleus	7.7922% (6/77)	0.4307% (18/4179)	5.01E-07
GO:0007059	chromosome segregation	11.6883% (9/77)	1.3640% (57/4179)	6.71E-07
GO:0045324	late endosome to vacuole transport	6.4935% (5/77)	0.3350% (14/4179)	3.28E-06
GO:0005200	structural constituent of cytoskeleton	7.7922% (6/77)	0.6222% (26/4179)	5.52E-06
GO:0031110	regulation of microtubule polymerization or depolymerization	5.1948% (4/77)	0.2154% (9/4179)	1.25E-05
GO:0008017	microtubule binding	6.4935% (5/77)	0.4786% (20/4179)	2.33E-05
GO:0003777	microtubule motor activity	3.8961% (3/77)	0.1436% (6/4179)	1.16E-04
GO:0030473	nuclear migration along microtubule	3.8961% (3/77)	0.1436% (6/4179)	1.16E-04
GO:0007020	microtubule nucleation	5.1948% (4/77)	0.3829% (16/4179)	1.64E-04
GO:0000132	establishment of mitotic spindle orientation	3.8961% (3/77)	0.1675% (7/4179)	2.00E-04
GO:0000741	karyogamy	3.8961% (3/77)	0.1914% (8/4179)	3.15E-04
GO:0008154	actin polymerization or depolymerization	2.5974% (2/77)	0.0479% (2/4179)	3.35E-04
GO:0003786	actin lateral binding	2.5974% (2/77)	0.0479% (2/4179)	3.35E-04
GO:0007119	budding cell isotropic bud growth	2.5974% (2/77)	0.0718% (3/4179)	9.93E-04

OSLOM analysis also identified a unique cluster in *S*. *bayanus* that did not appear in any simulation in any species suggesting a functionally unique complex involved in invasive growth in response to glucose limitation (GO:0004169 p = 1.41x10^-5^). In *S*. *cerevisiae*, galactose metabolism genes can only be induced by the presence of galactose, but in *S*. *bayanus* they are also induced in response to less preferred carbon sources such as ethanol, raffinose, sucrose and glycerol [62]. Combined, these results suggest that the unique vitality of *S*. *bayanus* under glucose limitation could involve both upregulation of galactose metabolism genes and the maintenance of a protein interaction cluster involved in invasive growth.

## Conclusions

In this paper, predicted PPI interaction networks were used to supplement evolutionary insights gained via traditional comparative genomic methods. We used the Protein-protein Interaction Prediction Engine (PIPE) to generate predicted interactomes for 5 closely related species of yeast. Through the use of a simulated null model, we provide strong evidence for conservation of PPIs throughout the yeast interactomes, with fewer than expected changes for about a quarter of the network. Changes in PPIs were not well predicted by sequence change, indicative of purifying selection on relatively small PPI interfaces. GO analyses allowed us to identify classes of proteins whose PPIs are conserved, that were not identified via sequence conservation alone, suggesting that additional insights are to be gained from analysis of PPI networks.

## Supporting information

S1 Table Summary of the GO terms present in the 4179 genes used in this study, obtained from Scannell *et al*. [18]. (XLSX)

S2 Table PIPE predicted protein-protein interactions in *S*. *cerevisiae*. (XLSX)

S3 Table PIPE predicted protein-protein interactions in *S*. *paradoxus*. (XLSX)

S4 Table PIPE predicted protein-protein interactions in *S*. *bayanum*. (XLSX)

S5 Table PIPE predicted protein-protein interactions in *S*. *kudriavzevii*. (XLSX)

S6 Table PIPE predicted protein-protein interactions in *S*. *mikatae*. (XLSX)

S7 Table Summary of the following statistics for each of the proteins studied: dN, dS, ω, degree, γ. dN, dS, and ω were estimated by Scannell et al. [18] under M0, "degree" is the number of PIPE predicted interactions for each protein in *S*. *cerevisiae* and γ is the inferred number of PPI changes for each protein across the 5-species tree.
(XLSX)

S8 Table Summary of the proteins, and the GO terms they are enriched for, with high or low ω, as well as high or low γ. (XLSX)

S9 Table Comparing the significant PPIs and their associated GO term enrichment between the four non-*cerevisiae* species. A significant PPI is defined as a PPI occurring in the wild type data but in none of the 100 respective simulations. These were filtered to find the unique significant PPIs for each species. GO enrichment analysis was carried out on the unique significant PPIs and then unique GO terms were identified for each set of PPIs.
(XLSX)

S10 Table Enriched GO terms unique to a given species’ set of significant interactions. (XLSX)

S1 File Clusters and GO enrichment identified for each species. (XLSX)
